# Impact of Iron Deficiency and Erythropoiesis-Stimulating Agents on Anemia in CKD Progression

**DOI:** 10.1155/ijne/2567637

**Published:** 2025-09-24

**Authors:** Collince Odiwuor Ogolla, Lucy W. Karani, Stanslaus Musyoki, Phidelis Maruti

**Affiliations:** ^1^Department of Applied Health Science, School of Health Science, Kisii University, P.O. Box 408-40200, Kisii, Kenya; ^2^Department of Medical Laboratory Science, School of Health Science, South Eastern Kenya University, P.O. Box 170-90200, Kitui, Kenya; ^3^Department of Medical Laboratory Science, School of Health Science, Kisii University, P.O. Box 408-40200, Kisii, Kenya

**Keywords:** anemia, chronic kidney disease, eGFR, erythropoiesis-stimulating agents, hemoglobin, iron deficiency, renal function

## Abstract

**Background:** Anemia is a frequent complication in patients with chronic kidney disease (CKD), with the incidence rising in stages 3–5. Iron deficiency and defective erythropoiesis are the major causes. Still, the role of iron status and the stimulating capability of ESAs on the progression of CKD have hardly been evaluated.

**Objective:** To assess the effect of iron deficiency and ESA therapy with respect to the correction of anemia and preservation of kidney function in patients with CKD stages 3–5.

**Methods:** A follow-up observational study was carried out in 120 CKD patients at nephrology department in a tertiary institution, from January 2023 to December 2024. The patients were classified into three groups: Group 1 and Group 3 considered iron-deficient, with no ESA and ESA therapy, respectively, while Group 2 was non–iron-deficient with no ESA. The parameters tested were hemoglobin levels, serum ferritin, transferrin saturation (TSAT), and estimated glomerular filtration rate (eGFR) at baseline and at 6 months after treatment. The ESA treatment given consisted of epoetin alfa or darbepoetin alfa, with iron supplementation given according to iron-deficiency status.

**Results:** Baseline hemoglobin levels were significantly lower in Group 1 (9.5 ± 1.2 g/dL), and these subjects were associated with a faster decline of eGFR by value per year (annual decline in eGFR: 3.5 ± 2.3 mL/min/1.73 m^2^) compared to Groups 2 and 3 (*p* < 0.01). The ESA-treated group (Group 3) exhibited relatively the greatest improvement in hemoglobin level (to 12.3 ± 1.5 g/dL) and the slowest decline in kidney function (1.7 ± 1.2 mL/min/1.73 m^2^). Iron supplementation produced greater changes in ferritin and TSAT.

**Conclusion:** Iron deficiency is a paramount modifiable driver of anemia and CKD progression. ESA treatment improves anemia and retards renal deterioration, especially when coupled with iron supplementation. Early detection and correction of anemia might merit interplay in pursuit of optimized CKD outcomes.

## 1. Introduction

Anemia represents a common and clinically relevant complication in chronic kidney disease (CKD) cases, especially in CKD stages 3–5. The estimated presence of anemia among advanced CKD patients stands between 50% and 60%. The mechanisms are decreased EPO production, iron deficiency, inflammation, and shortened erythrocyte lifespan [1, 2]. CKD anemia increases cardiovascular risks and leads to a faster kidney disease trajectory toward end-stage renal disease (ESRD), poor life quality, and increased all-cause mortality [3, 4].

In iron-related disorders, iron deficiency is paramount to anemic episodes in CKD and may occur as either absolute (depleted iron stores) or functional (inability to utilize stored iron due to inflammation or chronic disease) [5, 6]. In both types, iron is rendered unavailable for erythropoiesis, thereby impairing red blood cell production even when ESAs are administered [7]. The latest KDIGO 2021 guidelines state that iron status evaluation using serum ferritin and transferrin saturation (TSAT) is a must, both before and during treatment with ESAs [8]. ESAs are used in the general treatment of CKD anemia; however, their efficacy depends on sufficient iron availability. Landmark trials such as CHOIR, TREAT, and CREATE have revealed both the benefits and the risks of ESA therapy, making it important to tailor treatment strategies accordingly [9–11].

Recent findings have cemented the fact that there is a complex interplay between anemia and CKD anemia being not only a result of CKD but in fact acting as an exacerbator of the renal decline rate [12, 13]. Poorly controlled iron deficiency, especially its functional iron deficiency pattern driven by chronic inflammation, complicates treatment outcomes in CKD populations [14, 15]. Thus, disturbances in iron metabolism in CKD include hepcidin dysregulation, gastrointestinal malabsorption, and chronic blood losses, all impairing erythropoiesis [16, 17]. Despite the changing spectrum of ESA formulations, resistance in the untreated iron deficiency realm continues to be an important clinical challenge [18, 19]. Evolving treatment paradigms prefer individualized anemia management that powers iron repletion prior to or in parallel with ESA therapy so as to improve treatment response and avoid harmful effects [20, 21]. But most of the evidence stems from high-income countries, thereby limiting translational evidence available for resource-constrained settings. This presents a dire need to evaluate the real-world effectiveness of such interventions in low-income countries where CKD burden is growing disproportionately and access to standard care remains uneven [22, 23].

Notwithstanding the signs of improvement in the management of CKD anemia, much knowledge is still lacking, especially in regard to the synergistic effect of iron deficiency and ESA therapy on kidney function trajectory. Most of the previous studies have been on hemodialysis populations, and there are scarce data concerning predialysis CKD patients in low-resource settings. This study therefore intends to examine the influence of iron deficiency and ESA therapy on anemia correction and CKD progression among patients with stage 3–5 CKD, thus addressing a critical gap in both regional and global nephrology practice.

## 2. Methods

### 2.1. Study Setting

This prospective observational study was conducted at the Department of Nephrology, at a tertiary institution, between January 2023 and December 2024.

### 2.2. Study Population

CKD stages 3–5 patients attending the nephrology outpatient clinic were enrolled consecutively. Eligible participants were at least 18-plus years old and informed consent was taken.

### 2.3. Study Design and Study Period

The research consisted of a 24-month prospective observational cohort study. Patients were subclassified under anemia treatments for comparative analyses. This was, therefore, a prospective observational cohort study rather than a RCT. Hence, patients were categorized based on iron status at baseline, as well as on treatments ordered by the attending physician in routine care. No experimental intervention was allocated by the study investigators; thus, registration of a clinical trial would not have applied or been required for that matter. The study was compliant with STROBE guidelines for observational research [24].

### 2.4. Sample Size Determination

With an anticipated moderate effect size (Cohen's *d* = 0.5) in differences across the hemoglobin levels of the groups and an 80% level of power, with a significance of 5%, 40 patients in each group were denoted as the minimum.

### 2.5. Sampling Method

A consecutive sampling approach was used where all eligible participants who consented for the study were enrolled.

### 2.6. Inclusion and Exclusion Criteria

Inclusion criteria involved adult CKD patients with stages 3–5 with documented anemia (Hb < 12 g/dL for females, < 13 g/dL for males) and complete baseline data. Exclusion criteria involved patients with malignancy, active infection, recent blood transfusion (< 3 months), or those on dialysis at baseline.

### 2.7. Data Collection

Data collection was done through enrollment of patients during follow-up visits at the Nephrology Outpatient Clinic. Upon giving informed consent, baseline demographic data, medical history, and clinical parameters were collected using standardized patient case report forms (CRFs).

#### 2.7.1. Blood Sample Collection

Venous blood was aseptically drawn from the antecubital vein blood using sterile EDTA vacutainer tubes (for complete blood count [CBC]) and plain tubes (for serum iron studies) following standard procedure of phlebotomy. All blood collection took place in early morning hours (between 08:00 a.m. and 10:00 a.m.) to avoid diurnal variations, particularly in serum iron levels.

#### 2.7.2. Laboratory Analysis

Hemoglobin and CBC: Analysis was done through an automated hematology analyzer (Sysmex XN-1000, Sysmex Corporation, Japan). Quality controls were conducted by calibration using commercial control materials on a daily basis.

Serum ferritin: These were quantified by chemiluminescent immunoassay on the Roche Cobas e411 analyzer (Roche Diagnostics, Germany). Tests were run following the instructions of the manufacturer.

TSAT: Calculated from serum iron and total iron-binding capacity (TIBC). These were measured using a colorimetric method on the *Beckman Coulter AU480 analyzer* (Beckman Coulter, USA).

Renal function assessment: Serum creatinine was analyzed using the enzymatic method on the Beckman Coulter AU480. Estimated glomerular filtration rate (eGFR) was calculated using the CKD-EPI 2021 formula, standardized for age, sex, and race. All assays were conducted in an ISO-certified central hospital laboratory.

Anemia was defined according to the World Health Organization (WHO) [25] and KDIGO 2021 guidelines [25] as hemoglobin levels < 13 g/dL in males and < 12 g/dL in females [26]. Iron deficiency was classified as absolute (serum ferritin < 100 ng/mL and TSAT < 20%) or functional (ferritin 100–500 ng/mL with TSAT < 20%), consistent with KDIGO criteria. These thresholds were used to stratify patients into treatment groups at baseline.

### 2.8. Data Documentation and Follow-Up

All patients were followed at 3-month intervals for 6 months. At each subsequent visit, hemoglobin, iron indices (ferritin and TSAT), and eGFR were measured once more by the same methods to maintain consistency. These follow-up visits were also the times when adverse events or clinical status changes were recorded.

Data were entered into a password-protected database, with research staff in charge of the data entry, and accuracy was checked by a second reviewer; in cases of discrepancies, a review of the source documents ensued.

### 2.9. Data Analysis and Interpretation

Statistical analysis was performed using R Version 4.5.1. Continuous variables were expressed as means ± SD, and categorical variables were given as percentages and frequencies. ANOVA and chi-square tests were conducted to image group comparisons. The level of Pearson's coefficient was able to identify correlations between hemoglobin and annual eGFR decline. *p* value lower than 0.05 was considered statistically significant.

### 2.10. Ethical Approval and Consent to Participate

The study was approved by the Institutional Review Board (ISERC/KTRH/0771/23). Written informed consent was obtained from all participants. The study adhered to the principles outlined in the Declaration of Helsinki. As this was an observational cohort study, no clinical trial registration number applies.

## 3. Results

For the study, a total of 120 patients with CKD stages 3–5 were enrolled. Anemia was diagnosed in 65 patients (54.2%) according to the WHO and KDIGO 2021 guideline definition of Hb < 13 g/dL for males and < 12 g/dL for females. Patients were stratified into three groups based on iron status and ESA therapy: Group 1: iron-deficient, no ESA therapy (*n* = 40), Group 2: non–iron-deficient, no ESA therapy (*n* = 25), and Group 3: iron-deficient, treated with ESA and iron supplementation (*n* = 55). Iron supplementation in Group 1 was administered orally, whereas Group 3 received a combination of ESA therapy (epoetin alfa or darbepoetin alfa) and intravenous iron (iron sucrose), in line with KDIGO recommendations.

### 3.1. Baseline Characteristics

Table 1 shows the baseline characteristics across the three groups. The mean age across groups was similar (Group 1: 58.3 ± 10.4 years; Group 2: 60.1 ± 9.2 years; Group 3: 59.4 ± 11.5 years). A higher proportion of participants were male (54.2%) overall. Most patients were in CKD stage 3, with 70% in Group 1, 64% in Group 2, and 69% in Group 3.

### 3.2. Changes in Hemoglobin and Iron Parameters After 6 Months

Following 6 months of treatment: Group 1 (oral iron only) showed modest improvement in hemoglobin from 9.5 ± 1.2 g/dL to 10.2 ± 1.1 g/dL (*p*=0.02). Group 2 (no treatment) showed a nonsignificant change in hemoglobin (11.0 ± 1.6 to 11.5 ± 1.4 g/dL; *p*=0.07). Group 3 (ESA + IV iron) had the most significant increase in hemoglobin: 10.8 ± 1.3 to 12.3 ± 1.5 g/dL (*p* < 0.001). Serum ferritin and TSAT levels improved significantly in both Group 1 and Group 3. Group 3 showed a mean ferritin increase of +2.9-fold, consistent with the use of intravenous iron (Table 2).

### 3.3. Kidney Function and eGFR Decline

At baseline, the eGFR was similar in all groups (range: 45–47 mL/min/1.73 m^2^). After 6 months: Group 1 expressed a more severe decline in eGFR, from 45.3 ± 9.5 to 42.8 ± 10.3 mL/min/1.73 m^2^, with an annualized decline of 3.5 ± 2.3 mL/min/1.73 m^2^. Compared to Group 1, Group 2's decline was only 2.2 ± 1.5, whereas Group 3 was the least: 1.7 ± 1.2 mL/min/1.73 m^2^ (*p* < 0.01 across groups). Multivariate regression analysis showed that iron deficiency (*β* = −2.24, *p*=0.03) and absence of ESA therapy (*β* = −1.97, *p*=0.04) were independently associated with faster eGFR decline, after adjusting for age, gender, CKD stage, diabetes, and hypertension (Table 3).

### 3.4. Progression to ESRD

During the follow-up: 6 patients in Group 1 (15%) progressed to ESRD and required renal replacement therapy. In contrast, only 2 patients in Group 2 (5%) and 2 patients in Group 3 (3.6%) progressed to ESRD (*p*=0.02 between Group 1 and Group 3). This underscores a possible protective role of combined ESA and IV iron therapy in reducing ESRD risk among anemic CKD patients.

### 3.5. Correlation Between Hemoglobin and eGFR Decline

**Figure 1:**
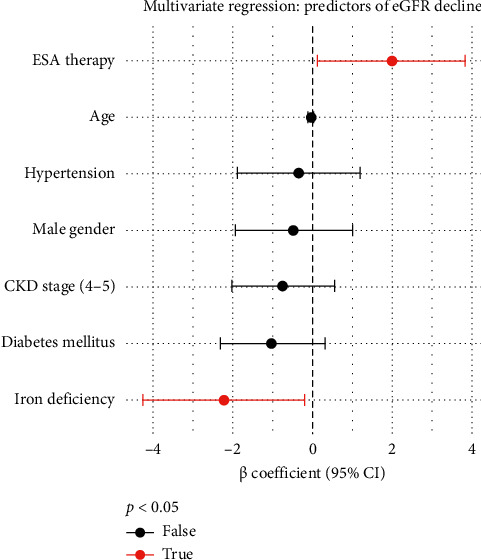
Multivariate regression analysis: predictors of eGFR decline over 6 months. Forest plot displaying the results of multivariate linear regression analysis assessing predictors of eGFR decline over 6 months among patients with CKD stages 3–5. Each point represents the β coefficient for a predictor variable, with horizontal lines indicating 95% confidence intervals. A vertical dashed line at zero represents the line of no effect. Predictors with statistically significant associations (*p* < 0.05) are shown in red, while nonsignificant predictors are shown in black. Negative *β* values indicate faster eGFR decline, while positive values indicate a protective effect on renal function.

**Figure 2:**
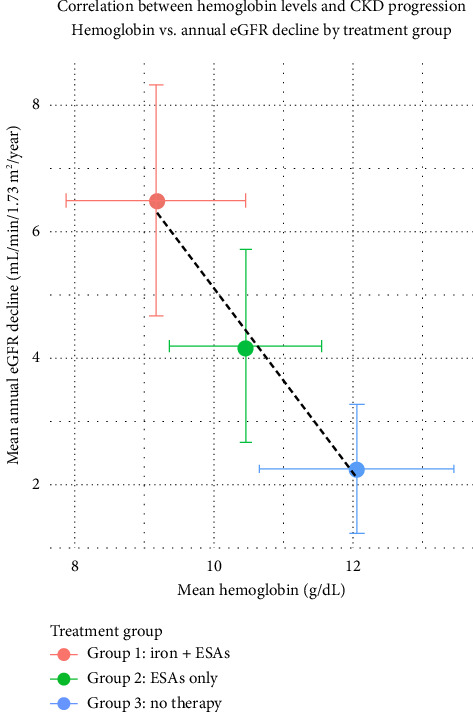
Correlation between hemoglobin level and eGFR decline in CKD patients. This scatter plot illustrates the relationship between hemoglobin concentration (g/dL) and eGFR (mL/min/1.73 m^2^) across all study participants (*n* = 120). Each point represents an individual patient. The regression line indicates a statistically significant positive correlation (*r* = 0.62, *p* < 0.001), suggesting that higher hemoglobin levels are associated with better kidney function. This supports the hypothesis that anemia severity is linked to the progression of chronic kidney disease. Data points were stratified by anemia management groups and analyzed using Pearson's correlation coefficient.

A moderate positive correlation was observed between final hemoglobin levels and eGFR at 6 months (*r* = 0.41, *p* < 0.01) (see Figures 1 and 2). Similarly, a negative correlation was found between TSAT and eGFR decline (*r* = −0.38, *p* < 0.01), indicating that better iron status was associated with slower CKD progression.

## 4. Discussion

The research looks into the relationship between anemia, iron supplementation, and erythropoiesis-stimulating agents and the progression of CKD. Our results demonstrates that there is a definite positive correlation between hemoglobin levels and eGFR indicating that lower hemoglobin values become associated with higher stages of CKD. In addition, patients receiving the concomitant therapies of iron supplementation and ESAs showed superior hemoglobin correction and slower decline in renal function relative to the other patients who received either one of the treatments or none.

Anemia is a common, multifactorial complication due to CKD but is mainly attributable to insufficient erythropoietin production, iron deficiency, and chronic inflammation [27, 28]. Our results align with the KDIGO 2021 guideline, recommending that anemia in CKD be treated on an individual basis, with particular regard for the use of intravenous iron and ESAs when there is clear evidence of iron deficiency or erythropoietin deficiency [8]. The improvement of hemoglobin levels in patients treated with therapy for iron deficiency and ESAs supports the larger clinical trials such as CHOIR [9], CREATE [10], and TREAT [11] that have shown that a modest hemoglobin target, that is, around 11–12 g/dL, can improve quality of life, reduce transfusion requirements, and perhaps slow down the progression of CKD when correctly imposed. However, these trials also warned against overtreatment because of cardiovascular risks associated with higher hemoglobin targets.

Our data fortify the concept of early correction of anemia in CKD patients, particularly with the combined treatment of iron supplementation and ESA therapy, in retarding CKD progression. Several studies strongly emphasize the ill effects of persistent anemia on renal hemodynamics, oxidative stress, and myocardial remodeling, which together accentuate renal decline and cardiovascular morbidity in CKD patients [29, 30]. The elevation in hemoglobin levels and lessening of eGFR decline occurring in our ESA-treated group accord with earlier reports that when anemia is adequately corrected, kidney function stabilizes, more so if iron stores are repleted beforehand [31, 32]. In addition, iron therapy alone, though mildly effective, may not perform satisfactory results in the moderate to severe range of anemia unless it is accompanied by ESAs, which emphasize the need for combination treatment [33, 34]. Our results suggest that this combined therapy can still be applied with clinical effectiveness if done judiciously with regard to iron status, even in the low-resource setting. Nevertheless, clinicians must exercise caution to avoid overtreatment. Evidence from studies such as CHOIR and TREAT has demonstrated that targeting hemoglobin levels above recommended thresholds may lead to higher rates of thromboembolic complications and cardiovascular mortality [35, 36]. Therefore, a balanced and individualized protocol, guided by current anemia management guidelines, is crucial to achieving optimal outcomes.

This study further strengthens the available evidence showing iron therapy, especially when combined with ESAs, as being effective in the clinical settings of a low-resource context. In contrast to the CHOIR and TREAT trials done in high-resource settings, our study was set to mimic routine clinical care in a public tertiary hospital, thereby enhancing the generalizability of the findings in similar contexts. We also noted that as advanced CKD is present, anemia prevalence significantly increases. This further backs the studies that anemia is not only a consequence of faster CKD progression but also precipitates it by way of tissue hypoxia, oxidative stress, and cardiac remodeling [37, 38]. In iron supplementation, the route of administration is crucial. While most patients in the study received oral iron, increasing amounts of evidence indicate that IV iron may be more effective in inflammation or advanced CKD when gastrointestinal absorption is impaired [39, 40]. Furthermore, by confirming the safety and efficacy of high doses of IV iron among dialysis patients, the PIVOTAL trial would still be regarded as a landmark [40, 41].

### 4.1. Clinical Implications

This study highlights the importance of diagnosing and correcting anemia in CKD early using treatment based on the available guidelines. Timely iron supplementation with appropriate assessment of iron status and judicious administration of ESAs can slow down renal function decline, enhance the patient's quality of life, and diminish the burden of transfusions.

### 4.2. Limitations

Several limitations matter in this study. First, it was a single-center observational study with quite a modest size, which may compromise external validity. Second, randomization was not done, and treatment groups followed available clinical practices, introducing potential confounding. Third, adherence to therapy and bioavailability of iron were not assessed, largely owing to the fact that predominance of oral iron limits interpretation on the comparative efficacy of different iron formulations. Randomized controlled trials in resource-limited settings are needed to evaluate the optimal combination and dosing regimen of iron and ESA therapy in CKD patients. Furthermore, long-term studies on cardiovascular outcomes and on the cost-effectiveness of these treatments must be undertaken.

## 5. Conclusion

In brief, this study shows that anemia is significantly associated with progression of CKD and that the combination of iron supplementation with ESA therapy has a more favorable outcome on the improvement of hemoglobin and stabilization of renal function than either treatment alone. Thus, in light of these findings, early management of anemia in CKD patients through a separate individualized approach must be emphasized according to available scientific evidence and based on local availability of resources.

## Figures and Tables

**Table 1:** Baseline characteristics of study participants.

Characteristic	Group 1 (iron-deficient, no ESA)	Group 2 (non–iron-deficient, no ESA)	Group 3 (iron-deficient, ESA + IV iron)	*p* value
Number of patients	40	25	55	—
Age (years), mean ± SD	58.3 ± 10.4	60.1 ± 9.2	59.4 ± 11.5	0.48
Male, *n* (%)	22 (55%)	13 (52%)	30 (54.5%)	0.93
CKD stage 3, *n* (%)	28 (70%)	16 (64%)	38 (69%)	0.88
Hemoglobin (g/dL)	9.5 ± 1.2	11.0 ± 1.6	10.8 ± 1.3	< 0.001
Serum ferritin (ng/mL)	50.4 ± 12.3	134.7 ± 24.1	48.9 ± 11.7	< 0.001
Transferrin saturation (%)	15.2 ± 4.5	26.5 ± 6.7	14.7 ± 5.0	< 0.001
eGFR (mL/min/1.73 m^2^)	45.3 ± 9.5	46.8 ± 8.7	46.0 ± 10.2	0.77
Diabetes mellitus, *n* (%)	18 (45%)	12 (48%)	24 (44%)	0.94
Hypertension, *n* (%)	36 (90%)	22 (88%)	50 (91%)	0.92

*Note:* At baseline, mean hemoglobin was significantly lower in Group 1 (9.5 ± 1.2 g/dL) compared to Group 2 (11.0 ± 1.6 g/dL) and Group 3 (10.8 ± 1.3 g/dL) (*p* < 0.001). Iron-deficient patients (Group 1) also had markedly reduced serum ferritin (50.4 ± 12.3 ng/mL) and transferrin saturation (TSAT: 15.2 ± 4.5%), indicative of absolute iron deficiency.

**Table 2:** Changes in hemoglobin, iron parameters, and kidney function after 6 months.

Parameter	Group 1 (oral iron)	Group 2 (no therapy)	Group 3 (ESA + IV iron)	*p* value (between groups)
Hemoglobin (g/dL)	9.5 ⟶ 10.2 ± 1.1 (*p*=0.02)	11.0 ⟶ 11.5 ± 1.4 (*p*=0.07)	10.8 ⟶ 12.3 ± 1.5 (*p* < 0.001)	< 0.001
Serum ferritin (ng/mL)	50.4 ⟶ 82.6 ± 20.5	134.7 ⟶ 137.2 ± 19.7	48.9 ⟶ 141.8 ± 26.4	< 0.001
Transferrin saturation (%)	15.2 ⟶ 19.1 ± 5.3	26.5 ⟶ 27.3 ± 5.1	14.7 ⟶ 26.9 ± 5.5	< 0.001
eGFR (mL/min/1.73 m^2^)	45.3 ⟶ 42.8 ± 10.3	46.8 ⟶ 44.6 ± 9.2	46.0 ⟶ 44.3 ± 9.8	0.02
Annualized eGFR decline	−3.5 ± 2.3	−2.2 ± 1.5	−1.7 ± 1.2	< 0.01
Progression to ESRD, *n* (%)	6 (15%)	2 (5%)	2 (3.6%)	0.02

*Note:* This table summarizes the changes observed over a 6-month follow-up period in hemoglobin levels, iron indices (serum ferritin and transferrin saturation), estimated glomerular filtration rate (eGFR), annualized eGFR decline, and progression to end-stage renal disease (ESRD) among the three study groups. Group 1 received oral iron only, Group 2 received no anemia therapy, and Group 3 received erythropoiesis-stimulating agents (ESA) in combination with intravenous (IV) iron. Values are expressed as mean ± standard deviation or number (%), with within-group *p* values representing statistical significance of change from baseline to 6 months. Between-group *p* values compare posttreatment values across all three groups using ANOVA or chi-square tests as appropriate.

**Table 3:** Multivariate regression analysis: predictors of eGFR decline over 6 months.

Predictor variable	*β* coefficient	95% CI	*p* value
Iron deficiency (yes vs. no)	−2.24	−4.29 to −0.19	0.03
ESA therapy (yes vs. no)	+1.97	+0.11 to +3.83	0.04
Age (per year increase)	−0.05	−0.12 to +0.02	0.14
Male gender (vs. female)	−0.48	−1.97 to +1.01	0.52
CKD stage (4–5 vs. stage 3)	−0.76	−2.05 to +0.53	0.24
Diabetes mellitus (yes vs. no)	−1.02	−2.34 to +0.30	0.13
Hypertension (yes vs. no)	−0.35	−1.89 to +1.19	0.66

*Note:* Multivariate linear regression analysis identifying independent predictors of eGFR decline over a 6-month follow-up period. The analysis adjusts for age, sex, CKD stage, diabetes mellitus, and hypertension. *β* coefficients represent the estimated annualized change in eGFR (mL/min/1.73 m^2^) associated with each predictor. Negative values indicate faster decline in renal function. Statistically significant predictors (*p* < 0.05) include iron deficiency and absence of ESA therapy, suggesting both are independently associated with more rapid progression of CKD.

## Data Availability

The data of the findings of this study are all shared in this article.

## Nomenclature

CKD: Chronic kidney disease
ESAs: Erythropoiesis-stimulating agents
RCT: Randomized control trial
ESRD: End-stage renal disease
